# Reduced Lentivirus Susceptibility in Sheep with *TMEM154* Mutations

**DOI:** 10.1371/journal.pgen.1002467

**Published:** 2012-01-26

**Authors:** Michael P. Heaton, Michael L. Clawson, Carol G. Chitko-Mckown, Kreg A. Leymaster, Timothy P. L. Smith, Gregory P. Harhay, Stephen N. White, Lynn M. Herrmann-Hoesing, Michelle R. Mousel, Gregory S. Lewis, Theodore S. Kalbfleisch, James E. Keen, William W. Laegreid

**Affiliations:** 1 U.S. Meat Animal Research Center, Agriculture Research Service, United States Department of Agriculture, Clay Center, Nebraska, United States of America; 2 Animal Disease Research Unit, Agriculture Research Service, United States Department of Agriculture, Pullman, Washington, United States of America; 3 U.S. Sheep Experiment Station, Agriculture Research Service, United States Department of Agriculture, Dubois, Idaho, United States of America; 4 Department of Biochemistry and Molecular Biology, School of Medicine, University of Louisville, Louisville, Kentucky, United States of America; 5 Great Plains Veterinary Educational Center, University of Nebraska, Clay Center, Nebraska, United States of America; 6 Department of Pathobiology, University of Illinois at Urbana-Champaign, Urbana, Illinois, United States of America; The Roslin Institute, University of Edinburgh, United Kingdom

## Abstract

Visna/Maedi, or ovine progressive pneumonia (OPP) as it is known in the United States, is an incurable slow-acting disease of sheep caused by persistent lentivirus infection. This disease affects multiple tissues, including those of the respiratory and central nervous systems. Our aim was to identify ovine genetic risk factors for lentivirus infection. Sixty-nine matched pairs of infected cases and uninfected controls were identified among 736 naturally exposed sheep older than five years of age. These pairs were used in a genome-wide association study with 50,614 markers. A single SNP was identified in the ovine transmembrane protein (*TMEM154*) that exceeded genome-wide significance (unadjusted *p*-value 3×10^−9^). Sanger sequencing of the ovine *TMEM154* coding region identified six missense and two frameshift deletion mutations in the predicted signal peptide and extracellular domain. Two *TMEM154* haplotypes encoding glutamate (E) at position 35 were associated with infection while a third haplotype with lysine (K) at position 35 was not. Haplotypes encoding full-length E35 isoforms were analyzed together as genetic risk factors in a multi-breed, matched case-control design, with 61 pairs of 4-year-old ewes. The odds of infection for ewes with one copy of a full-length *TMEM154* E35 allele were 28 times greater than the odds for those without (*p*-value<0.0001, 95% CI 5–1,100). In a combined analysis of nine cohorts with 2,705 sheep from Nebraska, Idaho, and Iowa, the relative risk of infection was 2.85 times greater for sheep with a full-length *TMEM154* E35 allele (*p*-value<0.0001, 95% CI 2.36–3.43). Although rare, some sheep were homozygous for *TMEM154* deletion mutations and remained uninfected despite a lifetime of significant exposure. Together, these findings indicate that *TMEM154* may play a central role in ovine lentivirus infection and removing sheep with the most susceptible genotypes may help eradicate OPP and protect flocks from reinfection.

## Author Summary

Ovine lentivirus targets the host immune system and causes persistent retroviral infections affecting millions of sheep worldwide. In primates, lentivirus resistance is attributed to mutant virus coreceptors that are not expressed. In sheep, some animals are resistant to lentivirus infection despite repeated exposure; however, the mechanism of resistance is unknown. We designed a genome-wide association study to test whether sheep might have genetic variation that protects against lentivirus infection. Our results showed that variation in an ovine gene (*TMEM154*) was associated with infection. Sheep with the ancestral type of this gene were nearly three times more likely to become infected than those with mutant forms. We also discovered two mutant forms predicted to abolish the protein's function. Although the biological function of *TMEM154* is unknown, our results indicate that it plays an important role in lentivirus infection in sheep. Producing sheep with the least susceptible form of *TMEM154* may help eradicate the ovine disease caused by lentivirus.

## Introduction

Visna/Maedi virus (VMV) and caprine arthritis encephalitis virus (CAEV) are small ruminant lentiviruses (SRLV) of the retroviridae family [1] that infect sheep and goats in major sheep producing countries worldwide. The exceptions are Iceland where VMV was eradicated after a 30-year effort [2], and Australia and New Zealand where VMV has not been reported in sheep but CAEV has been reported in goats [3], [4]. Once infected, seroconversion typically occurs within weeks to months and the infection is incurable. Sheep do not usually display signs of clinical disease in the first two years of infection. The first signs of disease are often loss of body condition and indurative mastitis (i.e., thin ewe syndrome and hard udder). When disease develops, severe clinical signs may include difficulty breathing, chronic wasting, loss of motor control, and arthritis. Ovine progressive pneumonia virus (OPPV) is a closely related North American counterpart to VMV and typically produces an interstitial pneumonia. Seroprevalence studies of U.S. sheep have shown that 36% of sheep operations have infected animals and 24% of all animals tested were seropositive [5]. The impact of subclinical OPPV infection is significant and includes detrimental effects on sheep production from breeding through weaning [6], [7], [8]. Considering that losses are cumulative during an animal's lifetime, the negative effects on ewe production and the sheep industry are substantial.

Natural transmission of ovine lentiviruses is primarily among adults, occurs most frequently after their first year [9], [10], [11], [12], [13], and is by the respiratory route [14], [15], [16]. In addition, some infections occur in lambs by ingestion of infected colostrum and milk [8], [17], [18], [19], [20], [21]. Ovine lentiviruses are macrophage-tropic but not T-lymphocyte-tropic and thus do not cause an immunodeficiency in sheep [22], [23], [24], [25], [26]. Persistence of ovine lentivirus in infected sheep is attributed to latent proviral DNA sequences integrated into the genome of a small fraction of monocytes circulating in the blood. Proviral DNA transcription and gene expression is suppressed until infected monocytes mature into macrophages as they migrate into the interstitial spaces of affected organs [27], [28]. Once in the target organs, infected macrophages initiate viral replication, which induces an inflammatory cascade that ultimately attracts more infected monocytes and other leukocytes. These lesions increase progressively, terminating in disease and eventual death.

Although there is no cure, the impact of disease can be reduced by lowering the prevalence. Voluntary SRLV control programs have been established in several European countries [29], [30], [31], [32], [33], [34]. OPP can be eradicated by testing and removing infected animals or by isolating lambs from seropositive dams at birth. The lambs are then raised on uninfected colostrum and milk, and maintained separately from seropositive sheep thereafter. Either of these methods may be used alone, or in combination, to break the cycle of transmission. However, an OPP-free flock is still susceptible to infection if exposed to other infected sheep or goats [35]. Thus, efforts to eradicate OPP and maintain infection-free status would be facilitated if replacement breeding stock were genetically resistant to lentivirus infections.

Examples of genetic resistance to lentivirus infection have been documented in human populations. Nearly all individuals who lack the lentivirus co-receptor CCR5 do not acquire human immunodeficiency virus (HIV) infection after significant exposure [36], [37], [38]. Moreover, an infected person receiving transplanted stem cells lacking CCR5 may be cured of HIV [39]. In the cases of VMV and OPPV, reports have suggested that host resistance to lentiviral infection may also occur in sheep [40], [41], [42], [43]. Significant breed effects on seroprevalence have also been observed in comingled flocks of purebred sheep, further indicating possible host genetic restriction [10], [12]. For example, in U.S. sheep the OPPV seroprevalence in purebred Finnsheep, Texel, and Suffolk was 77, 65, and 15%, respectively [12]. In Basque dairy-sheep, seroconversion was strongly associated with lifetime maternal VMV-serological status and was interpreted as evidence of genetic susceptibility [44].

The present article reports findings from a genome-wide association study (GWAS) that used naturally-exposed ewes, together with the International Sheep Genome Consortium SNP50 marker set, to test for genetic association with lentivirus infection. Ovine DNA sequence variation in a transmembrane protein gene (*TMEM154*) was associated with lentivirus infection. The ancestral *TMEM154* allele encodes a 191 amino acid polypeptide with glutamate (E) at position 35 and is associated with infection susceptibility. A mutant *TMEM154* allele encodes lysine (K) at position 35 allele and is associated with reduced susceptibility. Two deletion mutations were also observed in *TMEM154*, however there were not enough individuals with these deletions to test their effect. Together, these results suggest that *TMEM154* may play a central role in ovine lentivirus biology.

## Results

### Identifying matched pairs of OPPV infected cases and uninfected controls

**Figure 1:**
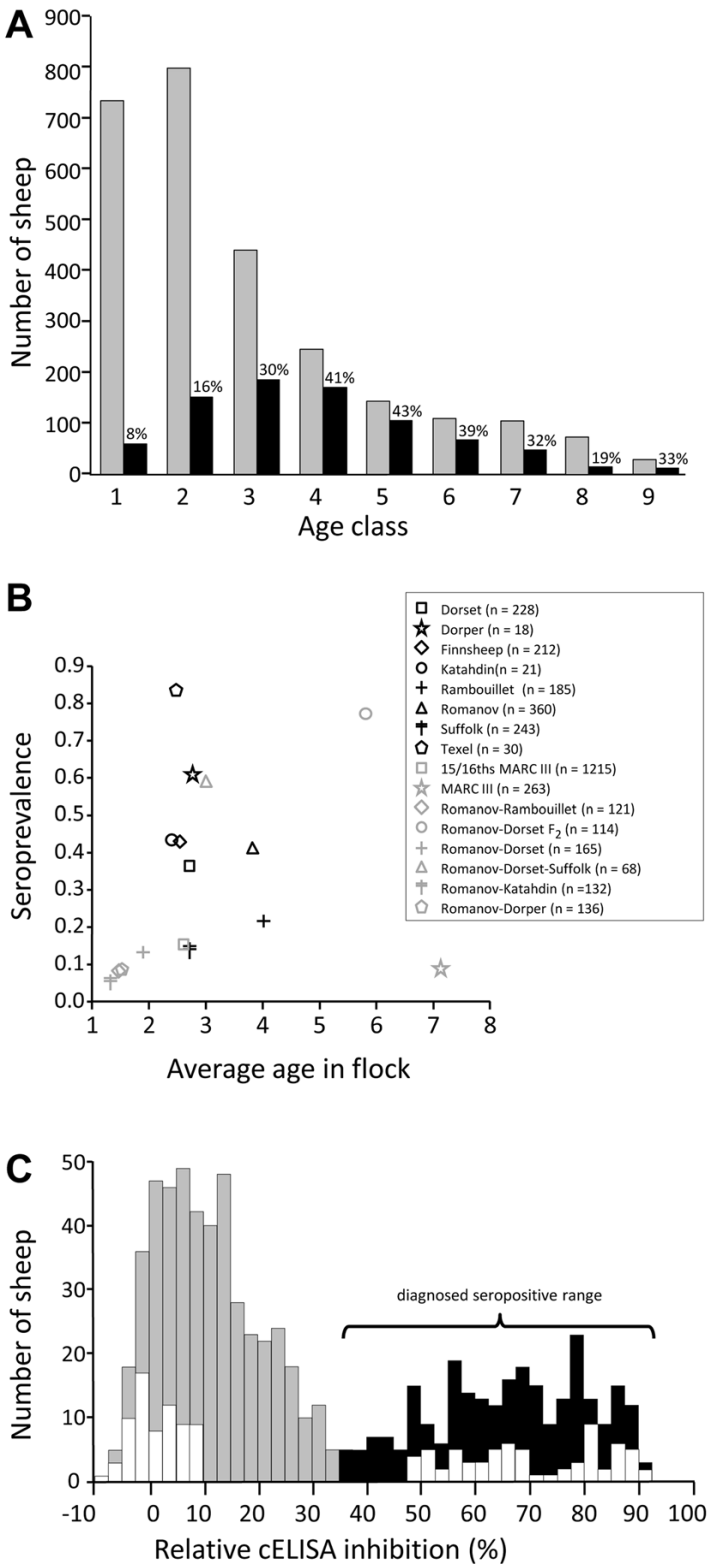
Seroprevalence of OPP in a research flock in 2003. Panel A, distribution of OPP serologic status by age class: shaded bars, OPP-negative; black bars, OPP-positive. The data include 3037 females and 508 males. Panel B, relationships between average seroprevalence, average age, and breed composition. Panel C, distribution of CAEV cELISA assay values in 736 ewes 5 years and older: shaded bars, OPP-negative; black bars, OPP-positive; white bars, sheep comprising 69 matched case-control pairs of 5- to 9-year-old ewes. The percent inhibition in cELISA assay values are those reported by the diagnostic testing laboratory and included negative values.

The presence of OPPV infection was tested with a competitive enzyme-linked immunosorbent assay (cELISA) in 3,545 breeding-age sheep from purebred and crossbred research flocks in South Central Nebraska, USA. This cELISA has high sensitivity (98.6%) and specificity (96.9%) in sheep naturally infected with OPPV [45]. Analysis by age class showed OPPV infection was lowest in 1-year-olds (8%), increased with age, and peaked at age 5 (43%, Figure 1A). From age 5 to 8 years, the number and proportion of OPPV-infected sheep declined in each year, indicating that the older infected sheep were leaving the flock at a faster rate than their uninfected flock mates. These results indicated that, by age 4, most sheep received sufficient OPPV exposure for infection to occur and that uninfected ewes appeared to have greater longevity in these flocks.

Although age is a risk factor for infection, seroprevalence varied widely within age class, depending on breed composition (Figure 1B). To examine the possibility that genetic risk factors may influence susceptibility to OPPV, matched case-control pairs consisting of infected and uninfected ewes were selected (Table 1). The strict matching criteria were intended primarily to reduce the variation in breed composition and OPPV exposure within each pair. The matching procedure identified 130 case-control pairs of 4- to 9-year-old ewes (Table 1). These pairs were used in a two-stage design with the goal of reducing falsely positive marker associations and minimizing the number of costly genome-wide scans. For the genome-wide association phase of the study, 69 pairs of 5- to 9-year-old ewes (white bars in Figure 1C) were evaluated first, while 61 matched pairs of 4-year-old ewes were held in reserve for verification of GWAS results.

**Table 1:** Historical attributes of matched pairs of infected and uninfected ewes.

Birth year	Age	Total sheep available	Matched pairs^a^	Average no. days between birth of pair	Average no. days between weaning of pair	Average no. of moves to different flocks	Average no. of months spent in flocks different from pair mate before collection	Suffolk pairs	Polled Dorset pairs	Rambouillet pairs^b^	Romanov pairs	Romanov-Dorset pairs	MARCIII pairs	Finnsheep pairs
1994	9	45	7	7.5	6.8	4.1	0	0	0	3	0	0	4	0
1995	8	94	9	8.5	4.0	3.6	0	0	0	5	2	0	2	0
1996	7	158	9	1.3	0.0	3.5	5	2	1	1	1	0	4	0
1997	6	184	19	1.9	0.0	4.5	1	1	1	1	0	4	11	1
1998	5	255	25	3.2	3.2	2.6	1	2	3	0	6	5	9	0
1999	4	422	61	1.4	1.2	2.4	14	3	1	6	11	8	30	2
Total	4 to 9	1158	130	na^c^	na	na	na	8	6	16	20	17	60	3

a All 130 pairs were matched for breed composition by comparing sire and dam line records. Each pair was birthed together in the same group of ewes in the same location over a 45-day period. In addition, all 260 sheep selected were born without difficulty, had a high level of vigor after birth, and were raised by ewes until weaning. The matching success rate for 5- to 9-year-old sheep with these criteria was approximately one matched pair per 8.9 sheep tested. Sibling relationships within pairs were analyzed retrospectively and identified two pairs of fraternal twins and 12 pairs of paternal half-sibs sharing on the sire side.

b These sheep were not born at USMARC but rather were purchased together as a group from a single Texas flock in May 1998.

c Not applicable.

### GWAS for OPP risk factors

**Figure 2:**
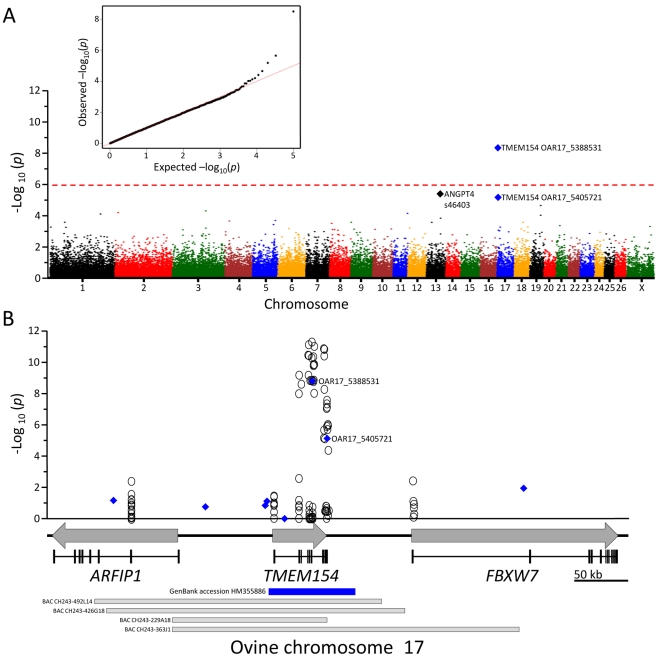
GWAS and fine-mapping of *TMEM154*. Panel A, relative genome position of 50,614 SNPs from the OvineSNP50 BeadChip and their unadjusted *p*-values for association with serologic status in 69 matched case-control pairs of 5- to 9-year-old ewes. Inset, Q-Q plot of the distribution of the test statistics. Panel B, relative genome position of 128 *de novo* SNPs identified in case-control sheep and their unadjusted *p-*values for association: circles, *de novo* SNPs; blue diamonds, SNPs from the OvineSNP50 BeadChip; blue bar, a 78 kb region of genomic DNA sequence containing the complete predicted *TMEM154* gene; grey arrows, predicted gene regions; vertical solid bars, predicted exons; horizontal shaded bars, ovine BAC clones. Oligonucleotides for *TMEM154* PCR and DNA sequencing are listed in Table S4.

Single nucleotide polymorphisms (SNPs) in the Ovine SNP50 BeadChip array (n = 54,241) were scored in 69 matched case-control pairs and tested for association with OPPV. The experimental design was estimated to have a detectable relative risk of genetic association that ranged from two to six in dominant and co-dominant models of inheritance, depending on marker allele frequency, and the extent of linkage disequilibrium (LD) between a marker and a disease allele (Materials and Methods). Of the 54,241 SNPs tested, 50,614 had quality scores in the acceptable range as determined by clustering and genotype calling algorithms. A single SNP on chromosome 17 had an unadjusted *p*-value of 3.19×10^−9^ (OAR17_5388531; Figure 2A). This was highly significant compared to the significance threshold of 1×10^−6^ (i.e., a significance level of 0.05 divided by 50,614). Moreover, the Quantile-Quantile (Q-Q) plot showed no evidence of an inflated test statistic caused by population structure. The c/t SNP OAR17_5388531 was in intron 5 of an ovine gene homologous to the human *TMEM154* gene on chromosome 4. The “c” allele of SNP OAR17_5388531 was on the sense strand of *TMEM154*, had a frequency of 0.257, and was associated with infected sheep. Another SNP (s46403) had the second lowest unadjusted *p-*value (2.22×10^−6^) and was on chromosome 13 in a gene similar to human angiopoietin 4 (*ANGPT4*). A third SNP (OAR17_5405721) had the third lowest unadjusted *p*-value (6.71×10^−6^) and was located in the 3′UTR of ovine *TMEM154*. The highly significant association of one SNP in ovine *TMEM154*, together with the third best SNP association being located in the same gene, suggested that a genetic risk factor associated with OPPV infection existed in this genomic interval. Subsequent efforts were directed towards characterizing the genomic region of ovine *TMEM154*, discovering additional polymorphisms, and testing them for association with infection.

### Ovine *TMEM154* DNA sequence assembly and SNP discovery

The complete sequence of the *TMEM154* region was not available for sheep and thus was determined by identifying and sequencing four overlapping bacterial artificial chromosomes (BACs) spanning approximately 400 kb. A contiguous 78 kb region was assembled *de novo* and appeared to contain the complete *TMEM154* gene region (Figure 2B, GenBank Accession HM355886). Other contigs from these BACs contained exons similar to human *ARFIP1* and *FBXW7*. The ovine genes appeared to be in the same orientation and approximate positions as those in reported for *ARFIP1*, *TMEM154*, and *FBXW7* on human chromosome 4 and cattle chromosome 17. Sanger sequencing of targeted genomic DNA fragments amplified by polymerase chain reaction (PCR) in the *ARFIP/TMEM154/FBXW7* region revealed 128 additional SNPs in the 69 pairs of matched 5- to 9-year-olds. However, SNPs associated with OPPV infection were observed only within the *TMEM154* gene (Figure 2B). The results indicated that *TMEM154*, and not flanking genes, was the likely source of the association.

### Analysis of ovine *TMEM154* haplotypes encoding polypeptide isoforms

**Figure 3:**
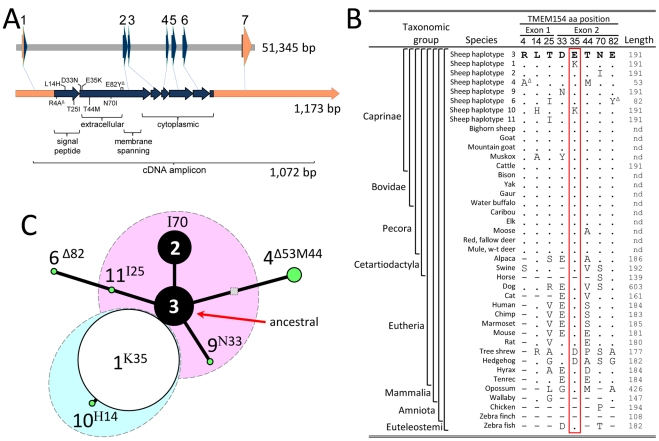
Physical maps, amino acid sequence comparison, and median-joining network of ovine *TMEM154*-encoded polypeptide isoforms. Panel A, genomic DNA and mRNA maps with missense SNPs and frameshift deletion polymorphisms: orange shading, 5′ and 3′ untranslated regions of exons; blue shading, coding regions of exons; grey shading, intron or intergenic regions. The codon polymorphism sequences are as follows: R4A^Δ^, **C**gc→[1 bp “c” deletion]gcg; L14H, ctt→cat; T25I, acc→atc; D33N, gac→aac; E35K, gaa→aaa; T44M, acg→atg; N70I, aat→att; E82Y^Δ^, gag→[7 bp “gagttta” deletion]tat. The signal peptide was predicted with SignalP 3.0 [60]; the extracellular, transmembrane, and cytoplasmic domains were predicted by comparison with human TMEM154 at www.uniprot.org. Panel B, a comparison of ovine non-synonymous *TMEM154* polymorphisms in exons 1 and 2 to those in related species. Polypeptide sequences for domestic sheep, bighorn sheep, domestic goat, mountain goat, muskox, cattle, gaur, yak, bison, water buffalo, caribou, elk, moose, red deer, fallow deer, mule deer, and white-tailed deer were deduced from Sanger sequencing of genomic DNA fragments amplified with ovine PCR primers. Amino acid sequences of the remaining species were obtained from Ensemble [61]. Symbols are as follows: dot, amino acid residues identical to those in sheep haplotype 3; dash, polypeptide region not present in, or not comparable to sheep; nd, not determined. Panel C, a median-joining network of *TMEM154* haplotypes encoding polypeptide isoforms [62]. The areas of the black and white circles are proportional to the haplotype allele frequencies in 5094 sheep. The symbols are as follows: pink and blue shaded areas, full-length *TMEM154* haplotypes with E and K at position 35, respectively; white circle, non-risk factor; black circles, risk factors; green circles, risk factor status unknown; shaded square, *TMEM154* haplotype predicted to have occurred but unobserved in the animals tested.

Although sequence variation in any number of gene elements can alter biological function, those that may directly affect the polypeptide sequence were evaluated first. The ovine *TMEM154* genomic assembly contained seven *TMEM154* exons encoding a 191 amino acid precursor protein (Figure 3A). The precursor protein contained a putative signal peptide at the N-terminus with a cleavage site predicted between positions 30 and 31 and resulted in a mature protein of 161 amino acids. The predicted mature ovine TMEM154 protein was 92.5, 67.3, and 53.8% identical with those of cattle, humans, and mice, respectively. The extracellular domain and signal peptide accounted for most of the amino acid sequence differences in these comparisons (83, 31, and 25% identity, respectively). The ovine intron/exon junctions were established by comparing the genomic sequences with those from a 1,012 bp reverse transcription (RT)-PCR fragment amplified from cDNA of contemporary animals. The ovine *TMEM154* mRNA and exon structure was similar to those reported for cattle, human, and mice (data not shown). Although RNA samples were not available for the 69 pairs of case-control sheep, the transcript sequence was determined for 11 case-control pairs of contemporary sheep and seven other available sheep. In all 29 sheep tested, the expected full-length transcripts were observed and their sequences corresponded to those from genomic DNA. Thus, alternatively spliced *TMEM154* transcripts did not explain the association observed with the SNP OAR17_5388531.

To evaluate whether amino acid sequence variation was encoded by ovine *TMEM154*, the exons were amplified from genomic DNA and sequenced for a panel of 234 animals that included all 69 matched case-control pairs and 96 rams representing common U.S. sheep breeds. In these 234 animals, five missense SNPs (T25I, D33N, E35K, T44M, N70I) and two frameshift deletion polymorphisms (R4A^Δ^, E82Y^Δ^) were observed in the predicted signal peptide and the extracellular domain (exons 1 and 2, Figure 3A). Conversely, nonsynonymous SNPs and frameshift polymorphisms were not observed in exons 3 through 7 in any of these sheep. *TMEM154* exons 1 and 2 were then considered as potential “hotspots” for coding polymorphisms, and these exons were sequenced for more than 5000 sheep from research populations, revealing one additional missense SNP (L14H). Combinations of the eight “coding” polymorphisms were observed on haplotypes encoding eight distinct precursor protein isoforms. Four haplotypes were predicted to encode full-length polypeptides with glutamate (E) at position 35 (Figure 3B, designated 2, 3, 9, and 11). The E35 allele was in strong LD with the “c” allele of OAR17_5388531 associated with infected cases (r^2^ = 0.98). Two haplotypes (designated 1 and 10) encoded full-length polypeptides with lysine (K) at position 35. The remaining haplotypes (4 and 6) had frameshift deletions predicted to cause premature termination of translation and loss of the putative membrane spanning and cytoplasmic domains of TMEM154.

Comparing polymorphic ovine TMEM154 amino acid residues with those in related mammalian species indicated that haplotype 3 was the most likely ancestral isoform in sheep. Thus, the ancestral ovine precursor protein isoform is inferred to be a 191 amino acid polypeptide with a negatively charged E35 residue. Haplotype comparisons between mammalian species also showed the E35 residue is highly conserved in mammals and the positively charged K35 residue of TMEM154 was not observed in other species analyzed (Figure 3B). A median-joining network of haplotypes encoding polypeptide isoforms of ovine TMEM154 showed that the two truncated isoforms were located on the distal branches of the tree (Figure 3C, haplotypes 4 and 6). Because the more recent haplotypes appeared to have evolved towards dysfunction and OPPV resistance, relationships presented in Figure 3C provide a framework for evaluating the potential role of *TMEM154*-encoded polypeptide isoforms in ovine lentivirus infection.

### Analysis of *TMEM154* haplotypes as risk factors for OPPV infection in matched cases and controls

We hypothesized that the more ancient full-length *TMEM154* haplotypes encoding E35 were genetic risk factors for OPPV infection because these alleles were in strong LD with the “c” allele of OAR17_5388531. Thus, sheep with one copy of haplotype 2 or 3 were compared to those without. Because the experimental design included paired samples, the McNemar's test for two correlated proportions was used for the analysis [46]. Also, the 61 matched pairs of 4-year-old ewes were deployed at this stage for comparison with results obtained from the 69 matched pairs of 5- to 9-year-old ewes. The dichotomous variable for this test was defined as having zero or one copy of the *TMEM154* genetic risk factor (i.e., haplotype 2 or 3). In one discordant type, the infected case had one copy of a *TMEM154* risk factor, but the uninfected control did not (Table 2 and Table S1). This type of discordant pair (n = 28) was consistent with the hypothesis. In the other discordant type, the infected case had zero copies of a *TMEM154* risk factor, and the uninfected control had one copy. This type of discordant pair (n = 1) was inconsistent with the hypothesis. In the 61 matched pairs of 4-year-old ewes, the odds ratio of these discordant pairs indicated that ewes with one copy of a full-length *TMEM154* E35 allele were 28 times more likely to be infected than those without (*p*-value<0.0001, 95% CI 5–1100). Compared to either SNP for OAR17_5388531 or E35K, the haplotype model for *TMEM154* yielded more significant results (Table 2). In this study, the predictive value of having one versus two copies of *TMEM154* risk factor alleles could not be determined because there were too few pairs of this type. Although an additive model was not excluded, one copy of the risk factor was significantly correlated with OPPV infection. If this were a diagnostic test for the 260 sheep in these 130 pairs, the predictive value (PV) for a positive test would be 85% with a sensitivity and specificity of 69% and 88%, respectively. These results were consistent with those of the GWAS and confirmed that *TMEM154* haplotypes encoding polypeptide variants were associated with OPPV infection.

**Table 2:** McNemar's test of TMEM154 polymorphims in matched case-control pairs of ewes.

	DNA marker								
	5388531			*TMEM154* E35K			*TMEM154* coding sequence		
Type of discordant pair (number of risk factors)^a^	69 pairs of 5- to 9- year-olds	61 pairs of 4-year-olds	130 combined pairs of 4- to 9-year-olds	69 pairs of 5- to 9- year-olds	61 pairs of 4-year-olds	130 combined pairs of 4- to 9-year-olds	69 pairs of 5- to 9- year-olds	61 pairs of 4-year-olds	130 combined pairs of 4- to 9-year-olds
Case (1), control (0)	36	30	66	36	30	66	41	28	69
Case (0), control (1)	2	2	4	2	2	4	0	1	1
Odds ratio	18	15	16	18	15	16	und^b^	28	69
Chi-square	29	23	53	29	23	53	39	23	64
*p*-value^c^	<0.0001	<0.0001	<0.0001	<0.0001	<0.0001	<0.0001	<0.0001	<0.0001	<0.0001
CI_95_	5–150	4–130	6–62	5–150	4–130	6–62	und	5–1100	12–2800

a The risk factor alleles were defined as the “C” allele for OAR_17_5388531, the E35 allele for the E35K variant, and haplotypes 2 and 3 for the TMEM154 haplotype variants. Haplotypes 2 and 3 were analyzed as equivalent risk factors. Animals without E35 or haplotypes 2 or 3 were scored as not having the genetic risk factor.

b Undefined because of the zero denominator.

c The p-value was calculated with McNemar's test with the continuity correction.

### *TMEM154* haplotypes 2 and 3 as risk factors for OPPV infection in multi-breed cohorts

Estimating the relative risk (RR) and PV of *TMEM154* haplotype alleles 2 and 3 provides an indication of the potential impact of full-length E35 alleles in other affected flocks. A total of 2705 sheep, 3-years and older, were evaluated in nine cohort studies to test *TMEM154* haplotype alleles 2 and 3 as risk factors for OPPV infection. These cohorts consisted of sheep from various breeds, ages, and production environments and were sampled over a span of seven years from research flocks in Nebraska and Idaho, and a private flock in Iowa. Animals were matched for gender and production environment in all cohorts; for three cohorts, animals were also matched for age at sampling. For each cohort, two-way contingency tables were used to analyze the relationship between the presence of *TMEM154* haplotypes 2 or 3 and being infected with OPPV (Table S2 and Table S3). For all cohorts, the combined RR of OPPV infection for animals with *TMEM154* haplotypes 2 or 3 was 2.85-times greater than those without (95% CI 2.36–3.43, *p*-value<0.0001). These cohort studies also confirmed that *TMEM154* haplotype alleles 2 and 3 are significant risk factors for OPPV infection in various breeds, and may be associated with lentivirus infection in multiple geographic locations and environments.

### Distribution of *TMEM154* haplotype risk factors in sheep populations

The frequency of *TMEM154* haplotype risk factors within breeds provides an indication of their potential susceptibility of OPPV in production environments similar to those described here. The combined frequencies of risk factor alleles 2 and 3 was highest in Texel (0.74) and lowest in Rambouillet (0.035, Table 3) and generally consistent with seroprevalence trends in the research flocks (Figure 1B). The most common truncated isoform of TMEM154 was encoded on haplotype 4, which was detected in Katahdin (0.15), Suffolk (0.13), Composites (0.033), Rambouillet (0.005), and Polypay (0.003). Overall, ovine *TMEM154* haplotypes encoding polypeptide isoforms 1, 2, 3, and 4 accounted for more than 99% of the haplotypes observed.

**Table 3:** Frequency distribution of ovine TMEM154 haplotypes encoding polypeptide isoforms.

				Breeds										
*TMEM154* haplotype	All sheep^a^	Sheep Diversity Panel v2.4	130 matched case controls 4- to 9-years-old	Columbia	Composites (MARCIII)	Dorper	Dorset	Finnsheep	Katahdin	Polypay	Rambouillet	Romanov	Suffolk	Texel
	(n = 5094)	(n = 96)	(n = 260)	(n = 208)	(n = 1254)	(n = 18)	(n = 74)	(n = 133)	(n = 36)	(n = 814)	(n = 541)	(n = 370)	(n = 180)	(n = 60)
1	0.77091	0.620	0.733	0.995	0.897	0.389	0.86	0.70	0.53	0.888	0.953	0.39	0.69	0.26
2	0.0822	0.21	0.11	-	0.065	0.361	0.14	0.20	0.04	0.031	0.006	0.08	0.16	0.51
3	0.125	0.15	0.12	0.005	0.003	0.250	-	0.102	0.25	0.071	0.030	0.54	-	0.23
4	0.017	0.02	0.03	-	0.033	-	-	-	0.15	0.003	0.005	-	0.13	-
6	0.0008	-	-	-	-	-	-	-	-	-	-	-	0.02	-
9	0.0004	0.005	-	-	0.0004	-	-	-	0.03	-	-	-	-	-
10	0.0022	-	-	-	-	-	-	-	-	0.006	0.006	-	-	-
11	0.0002	-	-	-	-	-	-	-	-	0.001	-	-	-	-
Risk factor (2 or 3)	0.2071	0.36	0.23	0.005	0.068	0.61	0.14	0.30	0.29	0.10	0.035	0.61	0.16	0.74

a Significant figures reported in allele frequencies are based on the number of haplotypes observed. Frequencies within a column adding up to other than 1.00 are due to rounding errors.

## Discussion

This report describes the discovery of an ovine gene that is associated with lentivirus infection in naturally-exposed U.S. sheep. In a GWAS with 50 k SNPs, one marker exceeded genome-wide significance and led to the identification of *TMEM154* haplotypes predicted to encode altered peptide sequences. *TMEM154* haplotypes 2 and 3 encode full-length polypeptides with E35 and appeared to be significant genetic risk factors for OPPV infection. Whether in matched pairs or cohorts, the presence of a *TMEM154* haplotype encoding a full-length E35 polypeptide was predictive of OPPV infection. The ovine *TMEM154* gene appears to be an OPPV susceptibility locus because the ancestral haplotype 3 was associated with infection. Thus, haplotype 1 (encoding a full-length K35 isoform) appears to be more recent and is associated with reduced susceptibility to OPPV infection. The two deletion mutations encoded on haplotypes 4 and 6 are also predicted to be more recent than haplotype 3 and indicate that *TMEM154* may be under selection for reduced function.

The function of the TMEM154 protein has not yet been reported for any species and remains unknown. In humans, the most abundant *TMEM154* mRNA was reported in CD19+ B cells and CD14+ monocytes with levels 15.8- and 7.6-fold above the *TMEM154* median, respectively (http://biogps.gnf.org). Expression of *TMEM154* in cells of monocyte lineage is of interest because they are the target cells for OPPV infection in sheep. It is plausible that mutant ovine TMEM154 polypeptide isoforms have altered function and decrease OPPV susceptibility. For example, the non-conservative substitution of K35 for E35 was associated with a decrease in OPPV susceptibility in homozygous individuals. The E35 residue in TMEM154 was highly conserved among the 32 Mammalian species tested; the only other substitution for E35 was the negatively charged aspartate (D) residue in hedgehog and hyrax (Figure 3B). Additional evidence that loss of TMEM154 function may reduce OPPV susceptibility is derived from the existence of two severely truncated polypeptides encoded by *TMEM154* haplotypes 4 and 6. Although there were not enough sheep with these haplotypes to test for association, the existence of two deletion mutations suggests that sheep without TMEM154 function may have a selective advantage when exposed to OPPV. The ovine TMEM154 protein does not appear to be essential for survival or reproduction because an 11-year-old purebred Suffolk ewe was identified in the Nebraska research flock with a homozygous genotype for *TMEM154* haplotype 4. Both the genomic and cDNA sequences of this ewe were consistent with her being a *TMEM154* “4,4 knockout” (data not shown). This ewe was kept year-round with OPP-infected ewes and lambed indoors in close quarters for 11 years, yet remained seronegative and in good condition prior to her death from an acute non-infectious illness. Multiple healthy *TMEM154* “4,4 knockout” rams have also been identified in the Nebraska flock. The identification of multiple animals with the *TMEM154* “4,4 knockout” genotype indicates that breeding natural *TMEM154* knockouts is possible and a functional *TMEM154* gene is apparently not required for sheep to thrive and reproduce.

### Limitations and strengths of the study

Ovine gene regions including *CCR5* and *DRB1* were previously associated with OPP provirus levels in candidate gene studies [41], [42], [43]. However, the OvineSNP50 BeadChip marker density and distribution were not sufficient for evaluating whether *CCR5* and *DRB1* gene regions were associated with OPPV infection in the present study. The nearest SNPs flanking *CCR5* (OAR19_55954161 and s65253) were approximately 20 kb from the coding region and had unadjusted *p*-values of 0.62 and 0.75, respectively. The degree of LD between the BeadChip SNP markers and those previously identified for CCR5 are unknown. The nearest SNPs flanking *DRB1* (OAR20_ 26932949 and OAR20_27259292) were greater than 100 kb from the coding region and had unadjusted *p*-values of 0.77 and 0.21, respectively. For either gene, there were no markers within 1 Mb that had unadjusted *p*-values less than 0.01. We acknowledge that a GWAS with 50 k SNPs and 69 case-control pairs limits the detectable genetic risk factors to those with large effects. Thus, other genetic risk factors for OPP may exist and were missed for lack of power in our study. In addition, it is not known whether *TMEM154* genetic risk factors are associated with specific OPPV strains such as those found in Nebraska, Idaho, and Iowa, USA. Strain differences, together with adverse production conditions like high animal density, indoor housing with poor ventilation, and moist climates, may enhance transmission and overcome any or all host genetic resistance. Thus, it is not known whether genetic variants of *TMEM154* will be useful predictors of OPPV infection under other management and/or environmental conditions.

The success of the present GWAS study was dependent on seven key features of the research design, without which the allelic association may have escaped detection. The first important feature was the availability of a serological test with good sensitivity and specificity for correctly classifying infection status. Serological results from duplicate blinded samples tested in two laboratories indicated that less than 2% of the animals were inconsistently classified in the initial round of testing. Second, the matched case-control design with older sheep was a key feature for reducing variation in the management conditions, environment, breed composition, and pathogen exposure. The use of older sheep increased the chances that sufficient natural exposure had occurred so that a high proportion of susceptible individuals could become infected. Third, it was important to have sufficient numbers of older sheep to assemble enough matched pairs to detect an association. Of the older sheep tested, only 22% met the matching criteria. Fourth, the relatively diverse breed composition of the research flocks increased the likelihood that an association observed in the 69 matched pairs was not limited to one breed. Fifth, public availability of the OvineSNP50 BeadChip was essential to progress beyond a functional candidate gene approach for discovering gene-phenotype associations. *TMEM154* had not previously been identified as a candidate gene in lentivirus biology for any species and thus would not have been considered. Sixth, the SNP marker spacing on the 50 k chip in the region of *TMEM154* was fortuitous because a GWAS may have missed the *TMEM154* association if the SNP density was lower or the distribution of SNPs happened to be less serendipitous. A higher density SNP chip would increase the chances of a marker SNP being in LD with a polymorphism that influences the trait of interest. A higher density SNP chip may also rule out the association of neighboring genes, and thereby narrow the region of focus. Seventh, *TMEM154* in the study populations had three common haplotypes encoding polypeptide isoforms, two of which formed a risk factor group with a large effect. This was previously unknown and was determined by the evolutionary history of *TMEM154* in these sheep. Nevertheless, this report demonstrates that a GWAS approach with 50 k SNPs and 69 matched case-control pairs was successful in sheep.

### OPP infection in sheep without *TMEM154* risk factor haplotypes

Although *TMEM154* haplotype risk factors 2 and 3 were strongly associated with OPPV infection, some animals without these haplotypes were also infected. For example, 36 of 139 sheep with a 1,1 diplotype were seropositive in the pairs of matched case-control sheep. This is consistent with the concept that host genetic resistance is conditional. Many factors may contribute to a virus overcoming host genetic resistance including: a high viral dose during an exposure event, a long duration of repeated viral exposures, viral genetic adaptation to host defenses, and multiple routes by which infection may occur. In the latter case, other host-encoded genes may play significant roles. Thus, comparing the relative level of resistance conferred by various *TMEM154* haplotypes, together with the identification of additional host genetic risk factors, will be important for developing flocks that are genetically resistant to lentivirus infections.

## Materials and Methods

### Ethics statement

Prior to their implementation, all animal procedures were reviewed and approved by the care and use committees at the United States Department of Agriculture (USDA), Agricultural Research Service (ARS) Meat Animal Research Center (USMARC) in Nebraska, the USDA, ARS, Sheep Experiment Station (USSES) in Idaho, and Washington State University in cooperation with the USDA, ARS, Animal Disease Research Unit (ADRU).

### Animal sample collection and serologic testing

The USMARC (Nebraska) sheep population was sampled in 2003 (n = 3545) and used to select 69 matched case-control pairs of 5- to 9-year-old ewes for the GWAS. The same population was also used to select 61 matched case-control pairs of 4-year-old ewes for analyzing *TMEM154* haplotypes as risk factors for OPP infection. Animals not used in matched case-controls were used in unmatched cohort studies for validation as shown in Table S2. Animals were not members of more than one group. The USMARC sheep population is a relatively diverse flock with more than ten breeds representing genetic diversity for traits such as fertility, prolificacy, maternal ability, growth rate, carcass leanness, wool quality, mature weight and longevity [47].

The USMARC population was sampled again in 2010 and used to select a cohort of 280 ewes, 4- to 5-year-old, and raised in similar conditions as those sampled in 2003. The purpose was to determine if the association of *TMEM154* haplotypes with OPP infection was reproducible in animals sampled seven years later.

The USSES (Idaho) sheep population was sampled in 2004 and 2008 and used to select cohorts of 309 and 365 mature ewes, respectively. The purpose was to determine if an association of *TMEM154* haplotypes with OPP infection was evident in another research flock that was geographically and historically distinct from the Nebraska flock. The USSES sheep population contains Columbia, Rambouillet, and Polypay breeds.

The private Polypay sheep flock (Iowa) was sampled in 2009 and used to select a cohort of 218 mature ewes. The purpose was to determine if an association of *TMEM154* haplotypes with OPP infection was evident in a commercial flock distinct from those in Nebraska and Idaho. This commercial flock was chosen based on its availability.

Whole blood samples for serum fractionation and DNA extraction were drawn from the jugular vein into S-Monovette serum Z and EDTA KE 9 ml syringes, respectively (Sarstedt, Newton, NC, USA). Laboratory diagnosis for OPP was performed at the Washington Animal Disease Diagnostic Laboratory (Pullman, WA, USA) with a Caprine Arthritis Encephaltitis Virus (CAEV) competitive-inhibition ELISA (cELISA). This CAEV cELISA is applicable for the detection of OPPV antibodies in sheep [45], [48]. Briefly, this assay uses a proprietary monoclonal antibody derived from the fusion of goat splenocytes and mouse myeloma cells (VMRD, Inc., Pullman, WA, USA). This antibody is conjugated to horeseradish peroxide and is used to compete with serum antibodies for the CAEV antigen bound to the microtiter plate. Additional testing for OPP was performed at USMARC and ADRU with CAEV cELISA kits, according to manufacturer's instruction (VMRD, Inc., Pullman, WA, USA).

### Statistical analysis

#### GWAS analyses

Sixty-nine pairs of ewes were selected from a total of 736 in the 5- to 9-year-old age class. The OPPV seroprevalence of the 736 ewes was 43%. In dominant and co-dominant models, our GWAS design had a detectable RR of genetic association that ranged from two to six with 69 paired case-controls, 50,000 SNPs, a false-positive rate (alpha) of 0.05, and a false-negative rate (beta) of 0.1 (simulation data not shown [49]). In a co-dominant model of inheritance with a disease prevalence of 0.43, the minimum detectable RR was less than 2 for marker allele frequencies between 0.15 and 0.50, and LD values between 0.7 to 1.0. In a dominant model of inheritance, the minimum detectable RR ranged from 2 to 6 for conditions similar to those above. There were not enough matched pairs in this design to detect GWAS of recessively inherited disease risk alleles. SNP genotypes for the OvineSNP50 BeadChip DNA samples were measured and scored at GeneSeek Inc. (Lincoln, NE, USA), according to manufacturer's instructions (Illumina, Inc., San Diego, CA, USA). For determining the number of SNPs that performed reliably with the set of 138 ovine samples, a GenCall score greater than 0.7 was used as a cutoff and was determined by clustering and genotype calling algorithms provided by the manufacturer (Illumina, Inc., San Diego, CA USA). All single SNPs were analyzed for association with infection using PLINK v1.07 software [50], as described: http://pngu.mgh.harvard.edu/purcell/plink/.

#### Cohort analysis

The combined relative risk was assessed in sheep cohorts using the glimmix procedure of SAS 9.2 (SAS Institute, Cary, NC). The serological status of OPP was fit as the dependent variable in a Poisson model with a log link to give unbiased estimates of relative risk and slightly conservative (broad) confidence intervals [51]. Breed and risk/nonrisk diplotype status were treated as independent fixed variables, where the risk was defined as the presence of at least one *TMEM154* haplotype allele 2 or 3. Age was included as a covariate and the cohort was treated as a random variable.

### Genomic DNA sequencing of ovine *TMEM154*

Ovine BACs predicted to contain *TMEM154* were identified from those mapped to the ovine draft genome sequence http://www.livestockgenomics.csiro.au/sheep/oar2.0.php). BACs were isolated from an arrayed 10–12× sheep BAC library (CHORI-243, [52]), cultured, and the BAC DNA was purified. The BACs were derived from the Texel ram used for the ovine genome sequencing project (USMARC animal no. 200118011). Pooled samples of the four BACs (CH243-492L14, 270 kb; CH243-229A18, 147 kb; CH243-363J1, 329 kb; and CH243-426G18, 279 kb) were sequenced by synthesis with conditions optimized for 600 bp read lengths, according to manufacturer's instructions (Roche Applied Sciences, Branford, CT, USA). DNA sequences were assembled *de novo* with Newbler software provided by the manufacturer and the contigs were evaluated and viewed with Consed [53]. Contig assembly made use of information and sequence available for cattle and sheep at the National Center for Biotechnology Information (NCBI) and International Sheep Genomics Consortium (ISGC), respectively. A 78 kb region of genomic DNA sequence containing the complete predicted *TMEM154* gene was assembled with 70 k reads and 25 Mb of sequence. Four large contigs were manually joined with information derived from ovine mRNA sequences, and the annotated 78 kb sequence was deposited in GenBank (accession number HM355886).

### Genotyping *TMEM154* by sequencing genomic DNA and cDNA

Ovine *TMEM154* exons were genotyped by Sanger sequencing of PCR fragments amplified from genomic DNA (Table S4). DNA extraction and genetic analyses were performed in a manner similarly to that previously described [47]. Briefly, a 1,000 bp PCR product containing each exon was sequenced in the 138 matched case-control sheep and 96 rams from a diverse panel of common U.S. sheep breeds (MARC Sheep Diversity Panel version 2.4) [47]. After scoring polymorphisms from these 234 sheep in all exons, a second round of nested PCR fragments were designed so that: 1) a 700 bp amplicon was fully nested within each previous 1,000 bp amplicon, and 2) the amplification primers for the 700 bp products did not bind to polymorphic sites discovered from sequencing the 1,000 bp on the genome (Table S4). The combined Sanger sequences from each animal were scored and recorded manually. More than 60 thousand tracefiles and 6.9 million genotypes from the present report are publicly available via the internet (http://cgemm.louisville.edu/USDA/index.html).

For mRNA transcript analysis, ovine blood (3 mL) was collected (Tempus Blood RNA tubes, Life Technologies Corporation, Carlsbad, CA, USA) and stored at −20°C prior to RNA extraction. Whole blood RNA was purified by centrifugation and filtration according to the manufacture's protocol (Tempus Spin RNA isolation kits, Life Technologies Corporation). RNA quantity and quality were determined spectophotometrically (ND-1000, NanoDrop Technologies, Inc., Wilmington, DE, USA; and Agilent 2100 Bioanalyzer (Agilent Technologies, Inc., Santa Clara, CA, USA). The complete *TMEM154* mRNA coding region was amplified by PCR from cDNA (SuperScript III One-Step RT-PCR System, Platinum *Taq* High Fidelity, Invitrogen Corporation, Carlsbad, CA, USA). The 25 µL reactions contained 1× of the manufacturer's reagent cocktail, 0.2 µM each of the sense and antisense primers (Table S4), 0.5 µL SuperScript III RT/Platinum *Taq* High Fidelity Enzyme Mix, and 30–50 ng of total RNA. Reaction conditions were the following: 1 cycle of cDNA synthesis at 55 °C for 30 minutes followed by pre-denaturation at 94 °C for 2 minutes; 40 cycles of PCR amplification at 94 °C for 15 seconds, 58 °C for 30 seconds, 68 °C for 1 minute; and 1 cycle of final extension at 68 °C for 5 minutes. As a control for DNA contamination and any putative *TMEM154* pseudogenes, duplicate sample reactions to those described above were subjected to PCR without preceding cDNA synthesis. Successful amplification of 1,012 bp fragments was monitored by gel electrophoresis. Amplicons were not observed in RT-PCR reactions lacking cDNA synthesis. Following an Exonuclease I digestion [54], *TMEM154* RT-PCR amplicons were sequenced with dye-terminator chemistry and separated by capillary electrophoresis (ABI 3730, PE Applied Biosystems, Foster City, CA, USA). The oligonucleotide primers for PCR and sequencing are listed in Table S4. Sequences were analyzed for polymorphisms and scored manually with Phred and Phrap [55], [56], Polyphred (version 6.10) [57] and Consed software [53].

An artiodactyl species panel of DNAs similar to that described previously [58] was sequenced to provide an estimate of the likely ancestral state of the polymorphic ovine *TMEM154* codons. This panel is composed primarily of species from the Pecoran clade, whose common ancestor dates to about 30 million years ago [59]. Oligonucleotide primers derived from ovine *TMEM154* genomic sequences were used in PCR assays to amplify exons 1 and 2 and PCR products for both exons were produced for the following species: Wyoming bighorn sheep (*Ovis canadensis*, n = 7), American plains bison (*Bison bison*, n = 7), Alaskan caribou (*Rangifer tarandus*, n = 7) Wyoming elk (*Cervus canadensis nelsoni*, n = 7), Texas exotic red deer (*Cervus elaphus*, n = 2), Texas exotic fallow deer (*Cervus dama*, n = 1), gaur (*Bos gaurus*, n = 2), domestic goat (*Capra hircus*, n = 4), Arkansas exotic water buffalo (*Bubalus bubalis*, n = l), Wyoming mule deer (*Odocoileus hemionus*, n = 7), Wyoming white-tailed deer (*Odocoileus virginianus*, n = 5), Wyoming mountain goat (*Oreamnos americanus* n = 8), and Alaskan and Wyoming moose (*Alces alces*, n = 8), for a total of 66 non-ovine artiodactyl individuals. To ensure that amplified DNA sequences were not derived from spurious ovine DNA, only those sequences with distinctive species-associated nucleotide differences were included in the analysis. Proteins encoded by Pecoran species were more than 95% identical to that encoded by ovine *TMEM154* haplotype 3.

## Supporting Information

Table S1 Distribution of *TMEM154* risk factors and diplotypes in matched cases-control pairs of ewes.
(XLSX)

Table S2 *TMEM154* haplotype risk factor analyses in cohort studies.
(XLSX)

Table S3 TMEM154 genotypes by serological status in matched case-control sheep (n = 260) and sheep in cohort studies (n = 2,705).
(XLSX)

Table S4 Oligonucleotides for ovine *TMEM154* PCR, RT–PCR, and DNA sequencing.
(XLSX)
